# Modifiable Risk Factors of Behavioral and Psychological Symptoms in People with Dementia

**DOI:** 10.1093/geroni/igab046.2441

**Published:** 2021-12-17

**Authors:** Shinae Seo, Meghan Mattos

**Affiliations:** 1 University of Virginia School of Nursing, Charlottesville, Virginia, United States; 2 University of Virginia, School of Nursing, Charlottesville, Virginia, United States

## Abstract

Over ninety percent of people with dementia (PWD) experience behavioral and psychological symptoms, known to increase burden on care providers and healthcare systems. The purpose of this integrative review was to examine the modifiable risk factors of behavioral and psychological symptoms of dementia (BPSD) at the individual, caregiver, and environmental levels. An electronic database search was performed using PubMed, CINAHL[EBSCO], Web of Science, and PsycINFO from 2010-2020. Search terms included “dementia” AND (“Behavioral” OR “Behavioural” OR “Psychological” OR “Neuropsychiatric”) AND “symptoms” AND (“Independent Living” OR “Community Dwelling” OR “Community Living” OR “Living at Home” OR “Ageing in Place.” The search yielded 1,121 articles, and 14 articles were included in this review. Among the 14 articles, there were 11 modifiable risk factors presented across the individual, caregiver, and environmental levels. Individual-level factors included the presence of affective disorder, low quality of life, and leisure dysfunction. The modifiable caregiver-level factors included relationship with PWD, frequency of contact, caregiver burden, distress, frustration level, caregiver as a resource for PWD, and quality of dyadic relationship. One environmental factor, the presence of pandemic disease (e.g., COVID 19), was identified. This review presents the modifiable factors that contribute to the varied symptoms and multi domains of BPSD. Further research is necessary to determine whether, and to what degree, interventions targeting individual, caregiver, or environmental risk factors may reduce BPSD for PWD, caregivers, and providers within the community setting.

